# Supplementary material for: Developmental toxicity from exposure to various forms of mercury compounds in medaka fish (Oryzias latipes) embryos
Source: PeerJ. 2016 Aug 23;4:e2282. doi: 10.7717/peerj.2282 (PMC5012308; doi:10.7717/peerj.2282)
Supplement: Table S1 [file peerj-04-2282-s001.docx]

| **Supplementary Table. Public health guidelines for mercury in drinking water and fish: Creating context and justification for this study.** | | | | |
| --- | --- | --- | --- | --- |
| **Mercury Form** | **Measure** | **Medium** | **Concentration** | **Source** |
| Inorganic Mercury | Maximum Contaminant Level (MCL) | Drinking Water | 0.002 mg/L | US EPA Maximum Contaminant level (MCL) ^1^ |
| Inorganic Mercury | Guideline Value | Drinking Water | 6 µg/L | WHO’s *Guidelines for drinking-water quality (4^th^ ed., Chapter 8 – Chemical Aspects* ^2^ |
| Organic Mercury | Guideline Value | Diet (day per person | 2-20 µg | WHO’s *Guidelines for drinking-water quality (4^th^ ed., Chapter 8 – Chemical Aspects* ^2^ |
| Organic Mercury | Mean Mercury Concentration | Fish and Shellfish | 0.012-0.417 ppm | Seafood Hg Database: Mercury concentrations in U.S. commercial seafood items (Stony Brook University) ^3^ |
| Organic Mercury | Mean Mercury Concentration | Fish and Shellfish | 0.003-1.45 ppm | US FDA Mercury Levels in Commerical Fish and Shellfish (1990-2010) ^4^ |
| Methyl Mercury | Mercury Concentration | Fish and Shellfish | 0-4.54 ppm | US FDA Mercury Concentrations in Fish: FDA Monitoring Program (1990-2010) ^5^ |
| Methyl Mercury | RfD | BMDL | 0.1 µg/kg | US EPA Reference Dose (RfD) for Chronic Oral Exposure to Methylmercury ^6^ |
| Total Mercury | Mercury Concentration | Fish and Shellfish | 0-3.005 ppm | US FDA Mercury Concentrations in Fish: FDA Monitoring Program (1990-2010) ^5^ |
| Total Mercury | Mercury Concentration | Medicinals | 0.1-0.5 g/day | Chinese Ministry of Heath; Pharmacopeia of China ^7^ |

^1^ <https://www.epa.gov/your-drinking-water/table-regulated-drinking-water-contaminants> ^2^ <http://www.who.int/water_sanitation_health/publications/dwq-guidelines-4/en/>
^3^ <https://knb.ecoinformatics.org/knb/metacat/knb.295.2/knb>
^4^ <http://www.fda.gov/food/foodborneillnesscontaminants/metals/ucm115644.htm>
^5^ <http://www.fda.gov/Food/FoodborneIllnessContaminants/Metals/ucm191007.htm>
^6^ <https://cfpub.epa.gov/ncea/iris/iris_documents/documents/subst/0073.htm>
^7^ Pharmacopeia of China. Beijing: People’s Press, pp1–586, 2005. <http://wp.chp.org.cn/front/chpint/en/>
